# Completeness of the operating room to intensive care unit handover: a matter of time?

**DOI:** 10.1186/s12871-021-01247-3

**Published:** 2021-02-05

**Authors:** Fabian Dusse, Johanna Pütz, Andreas Böhmer, Mark Schieren, Robin Joppich, Frank Wappler

**Affiliations:** 1 Department of Anesthesiology and Intensive Care Medicine, University Witten/Herdecke, Medical Center Cologne-Merheim, Ostmerheimer Str. 200, 51109 Cologne, Germany; 2 Department of Anesthesiology and Intensive Care Medicine, University Hospital of Cologne, Kerpener Str. 62, 50937 Cologne, Germany

**Keywords:** Patient handover, Hand‐off, Handover duration, ICU, Communication, Information loss, Patient safety

## Abstract

**Background:**

Handovers of post-anesthesia patients to the intensive care unit (ICU) are often unstructured and performed under time pressure. Hence, they bear a high risk of poor communication, loss of information and potential patient harm.

The aim of this study was to investigate the completeness of information transfer and the quantity of information loss during post anesthesia handovers of critical care patients.

**Methods:**

Using a self-developed checklist, including 55 peri-operative items, patient handovers from the operation room or post anesthesia care unit to the ICU staff were observed and documented in real time. Observations were analyzed for the amount of correct and completely transferred patient data in relation to the written documentation within the anesthesia record and the patient’s chart.

**Results:**

During a ten-week study period, 97 handovers were included. The mean duration of a handover was 146 seconds, interruptions occurred in 34% of all cases. While some items were transferred frequently (basic patient characteristics [72%], surgical procedure [83%], intraoperative complications [93.8%]) others were commonly missed (underlying diseases [23%], long-term medication [6%]). The completeness of information transfer is associated with the handover’s duration [B coefficient (95% CI): 0.118 (0.084-0.152), *p*<0.001] and increases significantly in handovers exceeding a duration of 2 minutes (24% ± 11.7 vs. 40% ± 18.04, *p*<0.001).

**Conclusions:**

Handover completeness is affected by time pressure, interruptions, and inappropriate surroundings, which increase the risk of information loss. To improve completeness and ensure patient safety, an adequate time span for handover, and the implementation of communication tools are required.

## Background

In most hospitals the transfer of critically ill patients between different units, such as the operating room (OR) and the intensive care unit (ICU), are routine procedures. Whenever care is handed over, however, patient safety relies on effective communication between care providers and a complete transfer of relevant information. Multiple studies demonstrated that poor handovers may result in medical errors and patient harm [1–6]. Patient handover during anesthesia care as a factor of patient safety and risk management has become an issue of growing interest [7]. At present, too often these handovers are unstructured and performed in a traditional ad hoc fashion that rarely provides an appropriate transfer of necessary information [8].

The potential risk of ineffective communication during handover, which may lead to medical errors and sentinel events, has been demonstrated [3–6]. According to the Joint Commission on Accreditation of Healthcare Organizations over 60 % of adverse events in health care could be traced back to communication failure between physicians [3, 9]. Major risk factors for ineffective handovers include the lack of standardized procedures, time pressure, interruptions, suboptimal surroundings, multitasking, inadequate feedback between sender and receiver, and the absence of safety culture [8, 10–12].

Transferring patients in the perioperative setting, including the OR, the post-anesthesia care unit (PACU), and the ICU, poses specific challenges. The members of the multidisciplinary team, such as anesthesiologists, surgeons, intensivists, and nurses, may focus on different aspects of care. Furthermore, post-operative patient transfers usually consist not only of a verbal handover but also of physical patient transfer between two different teams during which information loss may occur [13, 14]. Moreover, in the assumption to reduce handover time, multiple procedures are often in progress simultaneously.

As handover quality directly impacts patient safety, multiple efforts were made to improve communication skills, such as team training, standardizing of procedures and communication, and implementing cognitive aids, like checklists [15].

Recently, several studies investigated patient handovers and observed the transfers from the emergency medical service to the emergency department [16, 17] or the ICU [18], and from ICU to OR [19] or the general ward, respectively [20]. Others included only separate subgroups, like cardiac surgical [21] or pediatric patients [22, 23], or they focused on specific healthcare professionals, such as nursing staff [18, 24].

As there is only limited data available, this study aims to prospectively evaluate the post-operative handover completeness, as an aspect of quality, of critically ill patients from anesthesia to a multidisciplinary surgical ICU.

## Methods

This prospective observational study was conducted in 2014 in a teaching university hospital in Germany. The study was approved by the ethics committee of Witten/Herdecke University (No. 108/2011) according to the Declaration of Helsinki. Written informed consent was obtained from all patients prior to study inclusion. All included patients underwent elective surgery and were transferred from the OR or the PACU to the ICU postoperatively.

Patients under the age of 18 years and those who had surgery without general or regional anesthesia were excluded.

Over a ten-week-period during regular day shifts all post-operative patient handovers were prospectively observed, whenever a patient was transferred from the OR/PACU to the ICU. Observations took place at the patient transfer room. Documentation of all handovers between anesthesiologists and intensivists was performed by an independent single researcher, who did not engage in the situation or the conversation between the physicians. Patients, whose handovers were not observed entirely or who had incomplete charts, were excluded.

In preparation of this study, a 55 item checklist for data recording was developed based on a literature review on the quality of post-operative handovers, the hospital’s standardized anesthesia records, and a standardized patient questionnaire from the pre-anesthetic assessment. The items on the checklist represented those on the standardized anesthesia files. All information that was transferred during the handover was documented on this 3-part checklist, which was structured in a pre-, intra-, and post-operative section.

The pre-operative section contained the following: Patient’s characteristics (name, age), medical history; American Society of Anesthesiologists physical status classification (“ASA-score”); pre-existing conditions (cardiovascular, pulmonary, neurological, hepatic, renal, metabolic, infectious or muscular diseases; allergies); long-term medication; anesthesia-related risks; anatomical features and substance abuse.

The intra-operative items included: Performed surgical procedure; complications or changes during the procedure; type of anesthesia; airway management; catheters (intravascular, nerve block, urinary etc.); hemodynamic; infusions and transfusions; blood loss; antibiotic treatment; anesthesiological course and pain management.

The post-operative data contained the postoperative diagnosis, pain therapy, drains, and other specific features.

In addition, the duration of each handover (time from first until last verbal communication concerning the patient) as well as the number and the reason of interruptions also recorded.

The checklist was tested for applicability during a trial period. Moreover, all involved medical personnel of the Department of Anesthesiology and Intensive Care Medicine (including the ICU staff) was informed that an observation of post-anesthesia handovers would take place for study purposes. However, no information regarding the content or subject of the study was disclosed.

The information collected during the observed handovers was compared to anesthesia records and patient charts by the same investigator.

Collected data were directly transferred into a spreadsheet (Microsoft Excel® for Mac, 2011, Microsoft Corporation, USA), pseudonymised, and verified independently by a second investigator. This was followed by a descriptive analysis of quantities and percentages, as well as a linear regression analysis and comparison of means (ANOVA) using SPSS (SPSS Statistics 22, IBM Corporation, Armonk, NY, USA).

## Results

During the study period a total of 102 patient handovers were observed. Five handovers had to be excluded afterwards due to incomplete records. Thus, a total of 97 handovers were included in the study. Patient characteristics are shown in Table 1.

**Table 1:** Patients’ characteristics

**Age, years**				
Mean±SD	Min.	Max.		
59±17,9	20	94		
**ASA, n (%)**				
I	II	III	IV	V
0	26 (27)	46 (47)	18 (18)	1 (1)
**Surgical specialisation, n (%)**				
NS	TS	AS	VS	misc.
40 (41)	13 (13)	35 (36)	6 (6)	3 (3)
**Infectious status, n (%)**				
isolation				
18 (19)				

*SD* standard deviation, *ASA* American Society of Anaesthesiologists physical status classification, *NS* neurosurgery, *TS* trauma surgery, *AS* abdominal surgery, *VS* vascular surgery, *misc.* miscellaneous

The average duration of the handover was 2:26 min (range 0:15 min to 8:40 min). In 34 % of the observed cases the patient handover was interrupted at least once. Interruptions were caused by handling the patient in 52 %, by phone calls in 42 %, and by other reasons in 6 % of all included cases.

73 % of all handovers were conducted by resident physicians, 25 % by anesthesiology specialists, and one (1 %) handover was conducted by a senior physician and a caregiver, respectively. Recipients of all handovers were the ICU-physician and ICU nurse. Surgeons were not present.

**Fig. 1:**
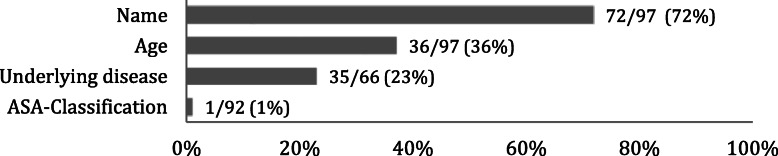
Percentage of the preoperative data documented and correctly verbally communicated during handover, *n* = observed number of cases ASA: American Society of Anesthesiologists

**Fig. 2:**
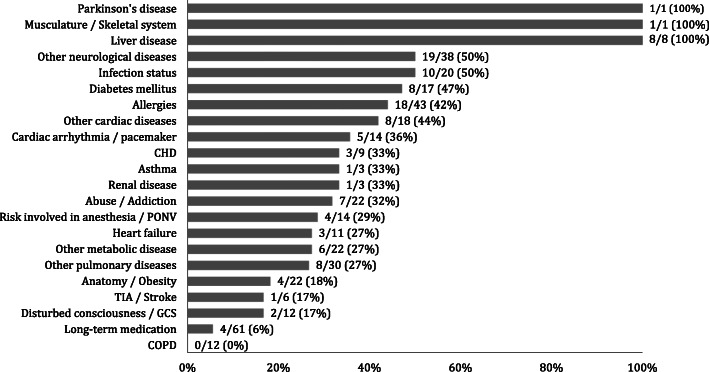
Percentage of the pre-existing diseases documented and correctly verbally communicated during handover, n = observed number of cases; CHD: Coronary Heart Disease; PONV: postoperative nausea and vomiting; TIA: transient ischemic attack; GCS: Glasgow Coma Scale; COPD: chronic obstructive pulmonary disease

**Fig. 3:**
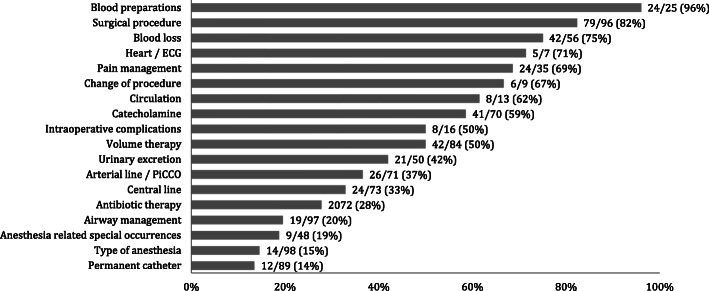
Percentage of the intra-operative data documented and correctly verbally communicated during handover, n = observed number of cases; ECG: electrocardiogram; PiCCO: Pulse Contour Cardiac Output

The results of the handover observations are presented in Figs. 1, 2 and 3. Results are presented as a percentage of correct and completely transferred patient data.

Regarding pre-operative information, the patient name was verbalized in 72 % of all cases and the age in 36 %. Primary diseases were transferred completely in 23 % of the cases (Fig. 1). Previous medication and allergies were communicated in 6 % and 42 %, respectively (Fig. 2). In 50 % of the cases, the infectious status of the patient was mentioned. Concerning the specific medical history, the data transferred correctly varies in a wide range between 0 % (chronic obstructive pulmonary disease [COPD]) to 100 % (Parkinson Disease, liver diseases, musculoskeletal disorder). In addition, it was noticeable that the long term medical history was rarely communicated (6 %), although data were available in the file in 63 % of all cases. Even the ASA-Score was mentioned only in 1 % of all cases.

Regarding intra-operative data (Fig. 3), information about the surgical procedure was ransferred correctly in 82 % of all cases. Though, in three cases (3 %) the transferred surgical details differed from the procedure that was documented in the patients’ chart. The type of anesthesia and airway management was communicated only in 15 % and 20 % of the cases, respectively. In contrast, information about intra-operative blood product administration was regularly mentioned (96 %).

The results of the post-operative data revealed that the rate of complete and correct information transfer was no more than 50 % over all four items. Pain therapy and diagnostics were communicated in 38 % and 41 % of all cases, respectively. Whereas special aspects were mentioned in 50 %, and information about drains were communicated least often (33 %).

**Fig. 4:**
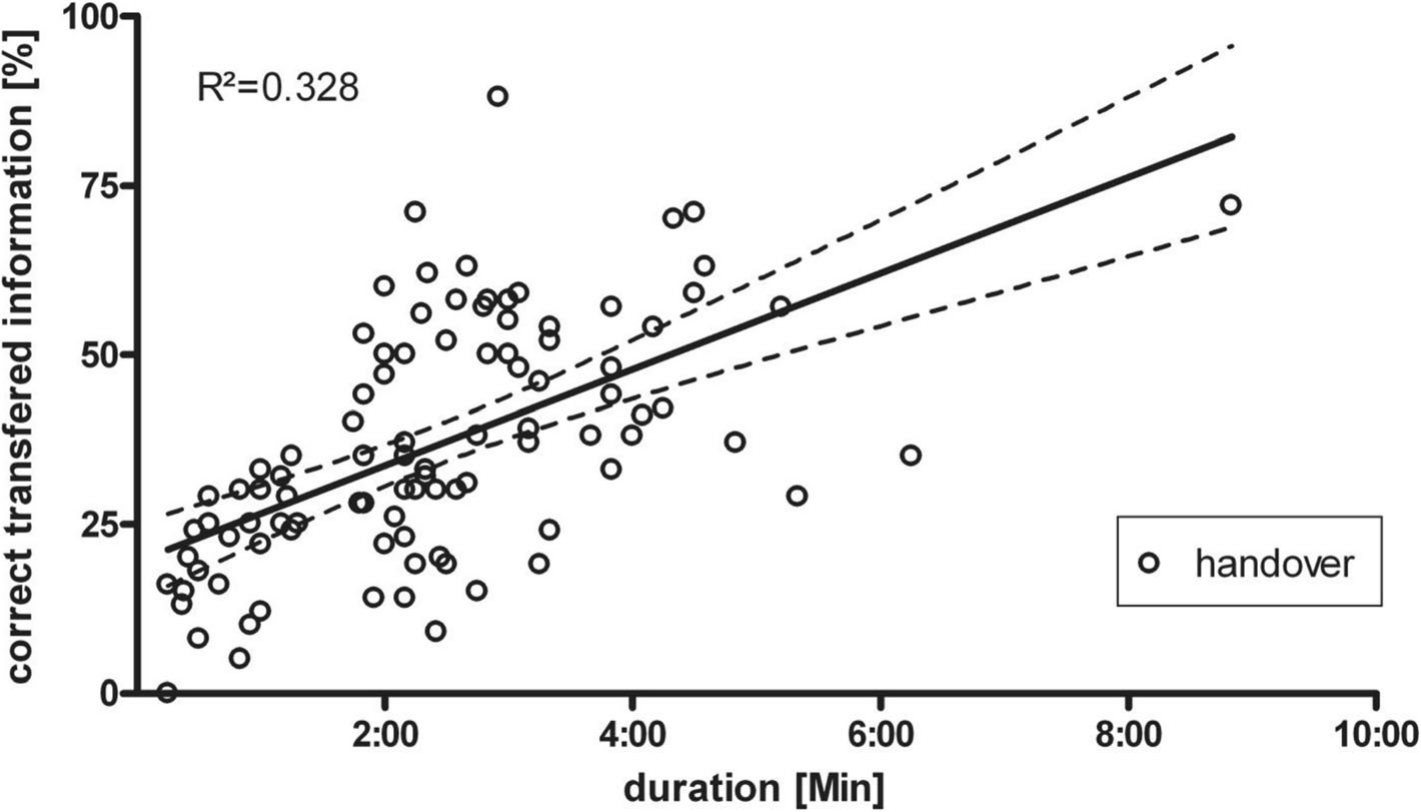
Dependency of handover completeness on duration. Data is shown as percentage of correctly transferred information (y-axis) against the duration of the respective handover (x-axis). Each circle represents one handover

A multivariate linear regression analysis including of the handover’s duration and the occurrence of interruptions revealed a significant relation between the handover’s duration and the percentage of correctly and completely transferred information (standardized ß coefficient 0.579, *p* < 0.001). Interruptions (phone calls, handling the patient, other) did not have any measurable significant impact on handover sufficiency (standardized ß coefficient − 0.010, *p* > 0.05). An univariate linear regression analysis calculating the relation of the handover’s duration on the completeness revealed a B coefficient of 0.118 with a 95 % confidence interval of 0.084–0.152 (Fig. 4).

**Fig. 5:**
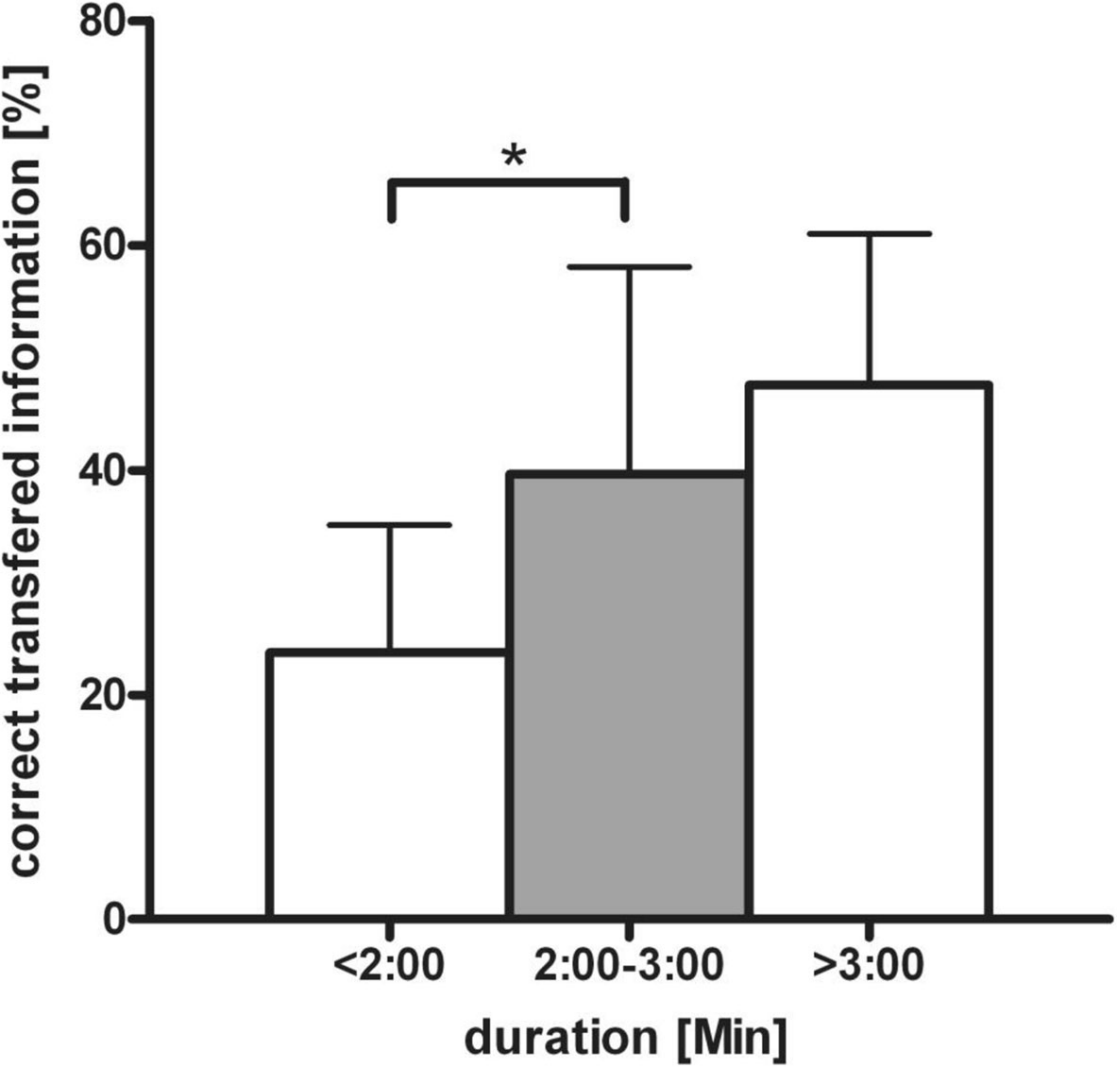
Comparison of means in groups according to the handover duration. *P < 0.001

Moreover, the variables were categorized into three groups according to the handover duration: handover duration of less than 2:00 minutes (d1, *n* = 33 handovers), 2:00 to 3:00 minutes (d2, *n* = 34), and more than 3:00 minutes (d3, *n* = 30). The comparison of means demonstrates a significant difference between group d1 and d2 (24 % ± 11.7 vs. d2 40 % ± 18.04, *p* < 0.001) but not between d2 and d3 (48 % ± 13.4) (Fig. 5).

## Discussion

The study demonstrates that an unstructured information management during handover of ICU patients has significant deficits. In many cases information is not communicated correctly by the anesthesiologists to the receiving ICU staff. These results underline findings of previous studies, which focused on several different clinical settings in which handovers of critical patients are performed frequently [2, 23, 25]. The fact that incomplete handovers with loss of information may contribute to adverse events and poor patient outcomes has been reported repeatedly [1, 15, 26]. However, patient safety can be easily increased by implementing structured handoffs, even with the aid of checklists or standardized protocols [26]. For instance, the introduction of a 19-item surgical safety checklist led to a decline of mortality and complications rates [27]. However, according to a recent study, the number of intraoperative handovers alone is not associated with the patient’s outcome [28], but this study did not consider the characteristics of intensive care patients and the specific challenges of post-operative transfers, which apparently bear an additional risk of losing important information.

Regarding patient safety, structured communication schemes, such as the SBAR (Situation, Background, Assessment, Recommendation), recommended by the world health organization (WHO) [3], as well as other schemes like SOAP (Subjective, Objective, Assessment, Plan), and I-PASS (Illness severity, Patient summary, Action list, Situation awareness and contingency plans, Synthesis by receiver) may lead to a higher quality of information transfer.

In anesthesiological practice, SBAR has been shown to improve the communication between professionals, enhance the safety climate and decrease the incidence of errors [29–31]. However, the use of a communication pattern alone does not guarantee a high quality of information transfer in critical care patients, as various factors may negatively affect patient handovers [32]. In the current study, it could be demonstrated that 33 of 97 (34 %) handovers were interrupted, in most cases by handling the patient, phone calls, or even private conversation. Multitasking [15], lack of time [33], as well as hectic und crowded circumstances are common causes for disturbed communication [34]. Thus, a calm atmosphere is needed for a focused handover. Even if an unpreventable interruption occurs, a handover checklist may help to resume to a structured communication without losing information.

In this study, 46 % of the handovers were performed by resident physicians. Thus, checklists may help less experienced anesthesia residents, who commonly performed the majority of handovers and are more prone for deficits in communication [25].

Transfers of peri-operative patients are often performed under considerable time pressure. The aim to reduce turnover time in the OR may be one of the main reasons why anesthetist’s handovers were typically brief and took place amidst a range of side activities [25, 33].

Interestingly, simultaneous handovers were just about 12 seconds faster than sequential handovers [11]. Therefore, multitasking during patient handoffs for presumed time saving purposes appears disputable. However, an “adequate time span” for a handover is difficult to define. In this study the duration of handover showed a wide range between 15sec and 8:40 min. Yet, an average of 2:26 min appears relatively short for a complete transfer of the patient’s clinical data, especially in comparison with the results of other studies [35, 36]. The completeness of a handover seems to be affected by its duration (Fig. 4). A longer handover time increases the likelihood that more information will be transferred. In particular, handover duration of less than two minutes is associated with a significantly increased risk of information loss (Fig. 5). The results of this study revealed that about one third of all observed handovers took less than 2:00 minutes. Such a brief time period can hardly suffice for an adequate transfer of information. On the other hand, the wide variation of handover completeness even in group d2 and d3 shows that a longer handover duration alone does not necessarily leads to a higher completeness.

To reduce handover time without affecting its quality, recently a handover protocol which includes Formula 1 pit stop and aviation models for quality and safety was developed. This protocol not only led to a reduction of handover duration, but also reduced the rates of technical errors and handover omissions [37]. This highlights the benefits of a structured handover protocol.

The informational content transferred between the involved physicians demonstrated a wide range (0–100 %). Common diseases and obvious facts are reported less frequently than less-common characteristics. A history of common neurological, cardiovascular, and metabolic disorders were reported in less than 35 %, however, information of liver failure and musculoskeletal diseases were transferred in 100 % of the cases. This may be due to the fact that the consideration of these common patients’ characteristics is part of the standard treatment on ICU and therefore is not necessarily regarded as an important fact by the reporting anesthetist. In addition, some information may not be transferred because the intensivist can take them just as well from the file, such as the long-term medication.

The presence of COPD has not been transferred in one single handover. This information, however, may require a specific post-operative therapy at an early stage, like NIV, or might, if unknown, result in problems of weaning from mechanical ventilation [38]. Similarly, missing information on the presence of a difficult airway or allergies can be life threatening in case of an intubation in the ICU or the administration of drugs.

Details about drains, diagnoses, and other specific surgical aspects were communicated correctly in less than 50 % of the cases. This highlights the need for surgeons to contribute to the post-operative handover, which has to be given at the patient’s bedside und conducted as a face-to-face conversation with the presence of four key providers, namely the delivering anesthesia provider and surgeon, the ICU physician, and the ICU nurse [39].

This study has limitations. Regarding previous studies which included 400 to 800 patients [2, 20, 23], the sample size of this study is relatively small. Nevertheless, the results revealed in the current study are representative for the researchers’ hospital and are in accordance with the literature.

The checklist used to observe the handovers was designed on base of the standardized anesthesia forms and literature review and has not been validated. For our study’s purpose, a complete checklist represents a perfect handover. The clinical impact of a “complete” handover on patient outcome, however, remains unclear.

Handovers of patients arriving from the OR and the PACU were not distinguished. Due to different time points, personnel, and care settings, handover completeness may have been affected.

Although the observer did not actively interact with the physicians, his physical presence alone may influence the manner in which the handover is carried out (Hawthorne effect) [40]. The observer had no specific training in observing techniques and no cross validation was performed. Furthermore, the observer only recorded whether an item of the checklist was mentioned or not. Handover content not included in the checklist, but also of importance to communicate, was not recorded. Though, the pertinence of the discrepancy between sent and received information during a handover can be relevant [32], it was not part of the study to investigate whether the transferred information has been understood.

## Conclusions

Unstructured patient handovers from post-anesthesia to ICU differ in quality and are often incomplete. Relevant information is lost, even though it may be of importance for the current treatment and the patient’s safety. The use of structured communication skills, specific checklists, and, in particular, an adequate time span could improve the handover completeness and thus the quality. Nevertheless, time pressure, interruptions, an outdated safety culture, and inappropriate cooperation among different disciplines remain issues to be solved in the future to make the transfer of critical patients as safe as possible.

## Data Availability

All relevant data is included in the manuscript. The Raw datasets used and analyzed during the current study are available from the corresponding author on reasonable request.

## List of Abbreviations

ASA: American Society of Anesthesiologists
CI: Confidence interval
CHD: Coronary Heart Disease
COPD: Chronic obstructive pulmonary disease
ECG: Electrocardiogram
GCS: Glasgow Coma Scale
ICU: Intensive Care Unit
I-PASS: Illness severity, Patient summary, Action list, Situation awareness and contingency plans, Synthesis by receiver
OR: Operating room
PACU: Post-anesthesia care unit
PONV: Postoperative nausea and vomiting
SBAR: Situation, Background, Assessment, Recommendation
SOAP: Subjective, Objective, Assessment, Plan
TIA: Transient ischemic attack
WHO: World health organisation
